# Photosynthetic rate prediction model of newborn leaves verified by core fluorescence parameters

**DOI:** 10.1038/s41598-020-59741-6

**Published:** 2020-02-20

**Authors:** Pan Zhang, Zhongxiong Zhang, Bin Li, Haihui Zhang, Jin Hu, Juan Zhao

**Affiliations:** 1 College of Mechanical and Electronic Engineering, Northwest A&F University, Yangling, Shaanxi 712100 China; 2 Key Laboratory of Agricultural Internet of Things, Ministry of Agriculture, Yangling, Shaanxi 712100 China; 3 Key Laboratory of Agricultural Information Perception and Intelligent Service, Yangling, Shaanxi 712100 China

**Keywords:** Light responses, Light stress

## Abstract

Due to the imperfect development of the photosynthetic apparatus of the newborn leaves of the canopy, the photosynthesis ability is insufficient, and the photosynthesis intensity is not only related to the external environmental factors, but also significantly related to the internal mechanism characteristics of the leaves. Light suppression and even light destruction are likely to occur when there is too much external light. Therefore, focus on the newborn leaves of the canopy, the accurate construction of photosynthetic rate prediction model based on environmental factor analysis and fluorescence mechanism characteristic analysis has become a key problem to be solved in facility agriculture. According to the above problems, a photosynthetic rate prediction model of newborn leaves in canopy of cucumber was proposed. The multi-factorial experiment was designed to obtain the multi-slice large-sample data of photosynthetic and fluorescence of newborn leaves. The correlation analysis method was used to obtain the main environmental impact factors as model inputs, and core chlorophyll fluorescence parameters was used for auxiliary verification. The best modeling method PSO-BP neural network was used to construct the newborn leaf photosynthetic rate prediction model. The validation results show that the net photosynthetic rate under different environmental factors of cucumber canopy leaves can be accurately predicted. The coefficient of determination between the measured values and the predicted values of photosynthetic rate was 0.9947 and the root mean square error was 0.8787. Meanwhile, combined with the core fluorescence parameters to assist the verification, it was found that the fluorescence parameters can accurately characterize crop photosynthesis. Therefore, this study is of great significance for improving the precision of light environment regulation for new leaf of facility crops.

## Introduction

In agriculture, photosynthesis research is important for understanding the physiological characteristics of crops and for predicting the degree of dry matter accumulation of crops^1–3^. A key question in agricultural research is how to precisely control the photosynthetic characteristics of crops based on their physiological characteristics. There is an obvious ‘old and new alternation’ behavior pattern in the growth process of crop canopy leaves^4^, when the new leaves of plant canopy develop into functional leaves, new newborn leaves will grow out and drive the whole plant to continue to grow. Therefore, the top newborn leaves of the plant play an important role in the growth and development of the entire crop. However, the leaves at the top of the canopy are generally newborn leaves, the photosynthetic apparatus is immature, the chlorophyll content is low and the photosynthetic capacity is also relatively low^5^. When there is excess light energy, the poor heat dissipation capacity of these leaves tends to cause light suppression, even light destruction, which in turn affects the growth of the whole plant^6–8^. The new technology of measuring chlorophyll fluorescence is mainly used for investigating photosynthetic mechanisms and for physiological research^9^. It is useful for accurately appraising the intrinsic characteristics of the photosynthetic apparatus, and also for accurately characterizing plant stress responses and resistance.

In recent years, many scholars have studied photosynthetic fluorescence characteristics and photosynthetic rate prediction models^10,11^. Zhang Yongguang *et al*. Zhang *et al*. estimated the photosynthetic capacity of the crop canopy using solar-induced fluorescence, which can be used to simulate regional primary production. A substantial improvement in the seasonal and spatial patterns of regional primary agricultural productivity can be achieved by constraining the equilibrium model of soil-canopy photosynthesis energy observations (SCOPE)^12^. Antal T *et al*. developed a chlorophyll fluorescence measurement system based on photosynthetic and fluorescence mechanisms to analyze fluorescence kinetic curves for continuous real-time monitoring of algal photosynthesis in photobioreactors^13^. Yin Gaofang *et al*. Yin *et al*. proposed a photosynthetic rate measurement method based on tunable pulsed light-induced fluorescence kinetics, using chlorophyll fluorescence as a probe in photosynthesis. They used the photosynthesis electron transport rate to evaluate the photosynthetic rate, which has high measurement accuracy. By studying the diurnal variation of chlorophyll fluorescence of six submerged plants^14^. Hong Shengjiang *et al*. Hong *et al*. found obvious changes in the maximum potential quantum efficiency. The diurnal periodicity and the diurnal variation of photon irradiance can also be considered when chlorophyll fluorescence is studied^15^. Duan Renyan *et al*. Duan *et al*. analyzed the fluorescence parameters of different growth stages of strawberries and found that there were some obvious differences between new, growing and senescent leaves^16^. Gao Yu *et al*. Gao *et al*. used chlorophyll fluorescence induction kinetic analysis of cucumbers varieties with different heat resistance. They found that in the process of preventing photoinhibition, the triple mechanism of reversible deactivation of the PSII reaction center, passivation of the oxygen release complex and heat dissipation plays an important role in heat shock prevention^17^. Zhang Haihui *et al*. Zhang *et al*. used chlorophyll convergence to construct a cucumber photosynthetic rate model and found that the accuracy of the model was improved using physiological factors^18^. Yin Jian *et al*. Yin *et al*. established a greenhouse tomato photosynthetic rate model using a wireless sensor network to collect environmental information, with the results showing good validity^19^. To date, the photosynthesis fluorescence characteristics of crops and construction of relevant photosynthetic rate models have been variously studied. However, the fluorescence parameters of new leaves and the intrinsic mechanisms of these leaves have not been considered. Therefore, it has become an important basis for efficient and precise regulation of facility crops to study the prediction model of photosynthetic rate of new leaves of crops with fusion fluorescence characteristics.

Cucumber was used as the experimental plant in this study, and photosynthetic and fluorescence data were obtained from newborn leaves using a multi-factorial test. Conventional environmental impact factors and core fluorescence parameters that had substantial effects on the photosynthetic characteristics of the newborn leaves were identified by correlation analysis. Furthermore, the BP neural network based on particle swarm optimization was used to construct the photosynthetic rate prediction model of the new leaves. At the same time, the core fluorescence parameters were used to verify the photosynthetic capacity of the newborn leaves, so as to lay a good theoretical foundation for canopy light supplement technology of facility crops.

## Results

### New leaf selection

**Figure 1:**
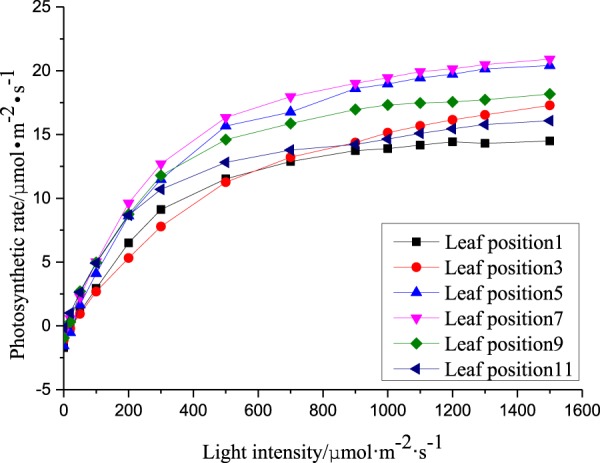
Photosynthetic rates of different leaf positions under 18 °C and 600 μmol·mol^−1^ CO_2_.

The photosynthetic rate response curves of different leaf positions of cucumber plants under different light conditions were obtained under constant temperature and CO_2_ (18 °C and 600 μmol·mol^−1^, respectively). The light response curves of different leaf positions are shown in Fig. 1, with the light intensity at the abscissa and the photosynthetic rate presented on the x and y axes, respectively.

There were obvious differences in the photosynthetic capacities between different leaf positions (Fig. 1). The top leaf (leaf position 1) has the worst photosynthetic capacity due to incomplete development of the photosynthetic apparatus. The 5–7 leaf positions are functional leaf positions, in which the photosynthetic apparatus is mature and the photosynthetic capacity is relatively optimal in comparison with other leaf positions in the plant. Although the leaves at position is 11 are old, the photosynthetic apparatus mature and intact and the chlorophyll content is relatively high. Therefore, the photosynthetic capacity at this position is better than at the top leaf. However, because the top leaf promotes longitudinal growth due to its phototropism, the new leaves of the crop leaves play an important role in the growth and development of the whole plant. Therefore, it is important to precisely construct a photosynthetic rate prediction model of these newborn leaves.

## Modeling Method Selection

### Comparison of model convergence

**Figure 2:**
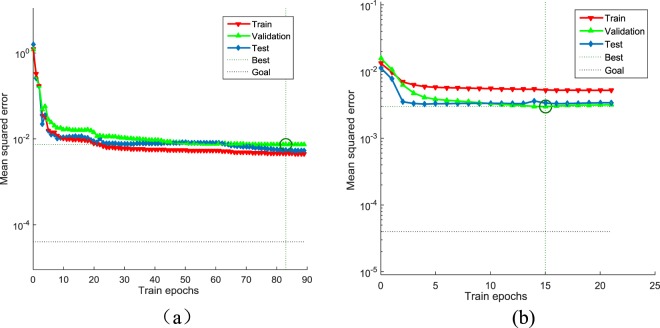
Iterative diagram of the BP and PSO-BP training processes: (**a**) BP network training and (**b**) PSO-BP network training.

The performance of convergence during the model training of the model determines the availability of the method. Therefore, with the temperature (Tem), CO_2_ concentration and light intensity (Par) as the input factors, the net photosynthetic rate as the output, to construct photosynthetic rate prediction model based on BP and PSO-BP and observe its convergence respectively (Fig. 2). Among them, the PSO-BP network only required 15 steps to reach the expected error level, no local flat zone or oscillations during the training process, and the mean square error was 2.9798 × 10^−3^. Although the BP neural network training process produced the desired error level with a mean square error of 7.3808 × 10^−3^, it required 83 steps and it had a local flat region that appeared during training. Therefore, the PSO-BP model overall had better convergence characteristics during the modeling process.

### Comparison of model accuracy

In order to further obtain the best photosynthetic rate modeling method, the mean square error, average absolute error, average relative error and determination coefficient R^2^ was calculated (Table 1). Compared with the BP method, the photosynthetic rate prediction model trained by the PSO-BP method has a significant improvement in accuracy.

**Table 1:** Comparison of the two different modeling methods.

Predictive model	Mean square error	Average absolute error	Average relative error	Decisive factor
BP	5.1874	0.1932	0.0315	0.9611
PSO-BP	2.3393	0.0459	0.1166	0.9867

It is found that the PSO-BP method not only had obvious advantages in terms of convergence but also had a significant improvement in accuracy during the construction of photosynthetic rate prediction models. Therefore, in this study, the PSO-BP method was used to construct a model for predicting photosynthetic rate of top newborn leaves.

### Fluorescence parameters prediction

As plant leaves use light energy mainly through three ways, (1) leaves use light energy for photosynthesis accounting for 97%, and the ETR is the most representative of this part of efficiency. (2) the excess light energy treated by the blade in the way of heat dissipation accounts for 2.5% of the total energy. (3) residual energy is released in the form of fluorescence. At the same time, the fluorescence parameters can correctly reflect the intrinsic characteristics of the photosynthetic capacity of crops. Therefore, based on the correlation analysis in Table 2 above and the strong correlation between environmental factors and ETR and NPQ before, pso-bp algorithm was used to construct fluorescence parameter prediction with environmental factors as input and ETR and NPQ as output respectively. The specific results are shown in Table 3. Among them, Model1 represents the photosynthetic rate prediction model, Model2 represents the ETR prediction model, and Model3 represents the qP prediction model.

**Table 2:** Correlation between each factor and the photosynthetic rate.

Variable	Tem	CO_2_	Par	qP	ETR	PhiPS2	Fv′/Fm′	NPQ	qN
Correlation coefficient	0.211^**^	0.317^**^	0.795^**^	−0.366^**^	0.920^**^	−0.453^**^	−0.458^**^	0.714^**^	0.019
Significant test value	<0.0001	<0.0001	<0.0001	<0.0001	<0.0001	<0.0001	<0.0001	<0.0001	0.744

Note: **Indicates a significant correlation at the 0.01 level (bilateral).

**Table 3:** Fluorescence parameters prediction.

	Predict target	Training set		Test set	
		R2	RMSE	R2	RMSE
Model1	Pn	0.987	0.05924	0.9867	0.05869
Modle2	ETR	0.9972	0.02913	0.9974	0.02739
Model3	NPQ	0.9819	0.04942	0.993	0.04178

According to the analysis in Table 3, ETR and NPQ, as the main influencing factors reflecting the intrinsic characteristics of photosynthetic ability, are found to have significantly better prediction effect on ETR and NPQ than those on photosynthetic rate under the premise of the same modeling method. Therefore, in the later construction of the photosynthetic rate prediction model, it can be considered to use fluorescence parameter as the target value to accurately characterize the photosynthetic capacity of crops.

## Discussion

The net photosynthetic rate(Pn) was positively correlated with Tem, CO_2_, Par, ETR, NPQ, and qN; and negatively correlated with qP, PhiPS2 and Fv′/Fm′ (Table 2). The first four of these factors are environmental, and the remaining factors are chlorophyll fluorescence parameters that are significantly related to the net photosynthetic rate (with the exceptions of qN). Based on the correlations between the environmental factors and photosynthesis, we found that the air temperature affects the activity of enzymes involved in photosynthesis, the crop transpiration rate and stomatal conductance^20,21^. These may in turn affect photosynthesis in crop plants. CO_2_ is the main substrate in plant photosynthesis and its concentration directly affects the accumulation of organic matter in photosynthetic carbon fixation^22,23^. Light intensity is the driving force of photosynthesis and is directly involved in the photoreaction phase of photosynthesis, providing sufficient electrons for the dark reactions and to ensure efficiency^24,25^. The light intensity, temperature and CO_2_ concentration in the greenhouse changed during the day, which greatly affected the crop photosynthesis and growth. Therefore, we included the environmental factors Tem, CO_2_ and Par in our model. In addition, analysis of the photosynthetic mechanism inside the leaves showed that the fluorescence parameters can accurately predict the photosynthetic capacity^26^. During photosynthesis, the qP measures the degree of closure of the reaction center, the ETR measures the rate of electron transfer during photosynthesis, and the PhiPS2 measures the actual initial light energy capture efficiency of the PSII reaction center with partial closure. The Fv′/Fm′ is the initial light energy capture efficiency of the open PSII reaction center, and the NPQ and qN measure the heat dissipation capacity when there is excess light energy. The correlation analysis in Table 2 shows that the ETR, NPQ and photosynthetic rate are significantly correlated.

**Figure 3:**
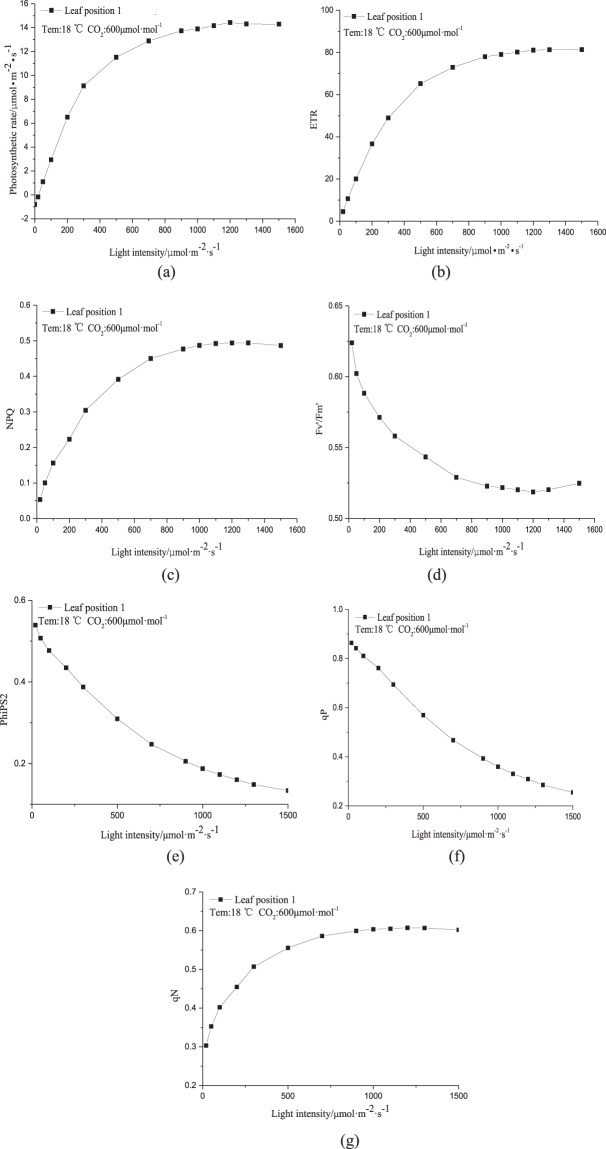
Photosynthesis and fluorescence response curves of the newborn leaves at 18 °C and 600 μmol·mol^−1^ CO_2_: (**a**) Pn response curve, (**b**) ETR response curve, (**c**) NPQ response curve, (**d**) Fv′/Fm′ response curve, (**e**) PhiPS2 response curve, (**f**) qP response curve, (**g**) qN response curve.

To further explore the relationship between fluorescence parameters and photosynthetic rate, the photosynthesis and fluorescence response curves were plotted under different light intensities (Fig. 3). The Pn, Fv′/Fm′, ETR, NPQ, qP, PhiPS2, qN and other photosynthesis and fluorescence parameters reflecting crop photosynthetic capacity under different light intensities were compared. The temperature and CO_2_ concentration were set to 18 °C and 600 μmol·mol^−1^, respectively; according to the fluorescence indices obtained earlier.

We can see from the Fig. 3, when the light intensity was in the range of 0–300 μmol·m^−2^·s^−1^, the photosynthetic rate of the new leaves increased with increasing light intensity. The intra-leaf ETR that is involved in photosynthesis also increased significantly, along with the NPQ and qN; while the Fv′/Fm′, PhiPS2 and qP were significantly reduced. When the light intensity was in the range of 300–1000 μmol·m^−2^·s^−1^, the photosynthetic rate of the new top leaves gradually decreased as the light intensity increased. The ETR inside the leaf is involved in the whole process of photosynthesis, and it gradually slows down as the light intensity increased. The ETR indicates that the leaf has begun to approach light saturation and that there is slight photoinhibition. The increase in the NPQ and qN also slowed down, indicating that the heat dissipation capacity of the leaf is close to its limit for dealing with excess light energy. The Fv′/Fm′, PhiPS2, and qP also trended downwards when the light intensity was in the range of 1000–1500 μmol·m^−2^·s^−1^. As the light intensity increased, the photosynthetic rate of the new leaves at the top position gradually flattened. The internal ETR of the leaf is involved in the entire photosynthesis process, and its increase is also mild. The NPQ and qN began to show a downward trend, suggesting that the leaves were beginning to be damaged by the strong light. The downward trends of the Fv′/Fm′, PhiPS2, and qP slowed down, indicating that the photosynthetic capacity was beginning to decay. The patterns of the ETR, NPQ response curves and photosynthetic rate response curves correlate well, with the ETR and NPQ highly correlated to the photosynthetic rate. Therefore, ETR and NPQ can be used for auxiliary verification of the photosynthetic rate model.

## Conclusion

The newborn leaves at the top of the cucumber play an important role in the growth and development of the whole plant; however, these leaves have an immature photosynthetic apparatus. Here, we constructed a photosynthetic rate prediction model using PSO-BP. Our findings may be applied to all vine crops, to allow the construction of precise newborn canopy leaf regulation models and the systematic study of the different growth stages in crops. Specifically, our conclusions are as follows:

Using the photosynthesis and fluorescence data obtained from our experiments a correlation analysis method was used to identify the main environmental factors (Tem, CO_2_ concentration and Par) and the core fluorescence parameters (ETR and NPQ) that influence the newborn canopy leaves. By comparing photosynthetic rate(Pn) response curve and fluorescence(ETR, NPQ, Fv′/Fm′, PhiPS2, qP, qN) response curve, it was found that the fluorescence characteristics in the photosynthetic process of crops had a better photosynthetic evaluation effect than the changes in the photosynthetic rate. Therefore, In the later research, we can use the fluorescence mechanism inside the leaves as the auxiliary reference standard of photosynthetic capacity, which can lay a good foundation for the accurate regulation of the light environment of the new leaves in the canopy.

Using the traditional BP and PSO-BP neural networks with the input terms Tem, CO_2_ concentration and Par as the inputs and net photosynthetic rate as the output, we constructed a model of the photosynthetic rate of the newborn leaves at the canopy. Our results showed that the PSO-BP photosynthetic rate prediction model was very precise, with a coefficient of determination R^2^ of 0.9947.

## Materials and Methods

### Experimental materials

The greenhouse experiment was conducted from October to December 2017 at the demonstration station base of the Comprehensive Service Area of Vegetable Industry in Jingyang, Xianyang, Shanxi. The cucumber variety “Bonai 14-3” was selected for the experiments. During breeding, full cucumber seeds were selected for soaking, germination and then cold treatment. A 50-hole nutrient mesh (540 × 280 × 50 mm) was used to support the seedlings and culture medium with an organic matter content of at least 50%, a humic acid content of at least 20% and a pH of 5.5–6.5 was used for seedling growth. Uniform irrigation, fertilization and illumination conditions were used during seedling growth. When the cucumber seedlings were sufficiently large, they were transplanted into the Base 2 East Sunlight Greenhouse, and then the net photosynthetic rate and fluorescence parameters of the newborn leaves were measured until the cucumber seedlings reached the flowering stage. No pesticides or hormones were used during the experiments and normal greenhouse conditions were applied.

**Figure 4:**
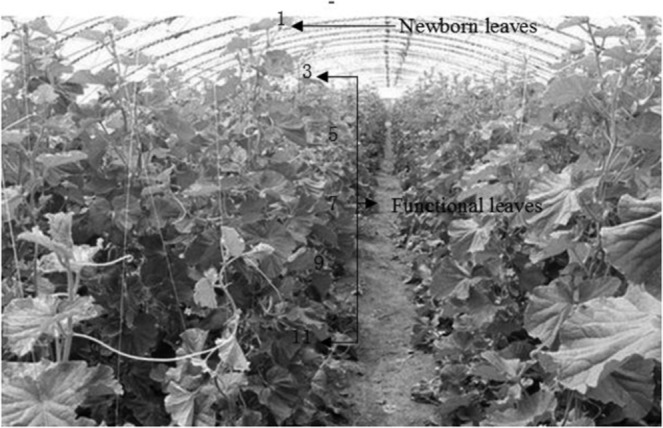
Positions measured on the plants.

Sixty strains of cucumber plants that exhibited good growth were selected for the experiments. Newborn cucumber leaves were used for the measurements, as shown in Fig. 4. Multi-environment factor testing was carried out using an LI-6800 portable photosynthesis instrument (LI-COR, USA) to measure the net photosynthetic rate(Pn) of the newborn leaves. The photochemical quenching coefficient (qP), photosynthetic electron transport rate (ETR), PSII actual photochemical quantum yield (PhiPS2), PSII effective photochemical quantum yield (Fv’/Fm’) and non-photochemical quenching coefficient (NPQ, qN) were recorded. The environmental conditions in the facility varied widely during the day, therefore, the temperature, CO_2_ concentration and light intensity were obtained by correlation analysis (Table 2). Because these parameters greatly affect photosynthesis, they were optimized for the experiments and for the model construction using a photosynthesiser self-tape module^27^. The temperature control was set to six different temperatures: 18, 20, 24, 28, 32 or 36 °C; and the CO_2_ injector was set to four different CO_2_ volume ratios: 300, 600, 900 or 1200 μmol·mol^−1^. When the light intensity is 0, the qP, ETR, PhiPS2, Fv’/Fm’, and NPQ are all invalid values, and the light saturation point of the cucumber leaves is approximately 990 μmol m^−2^·s^−1^. Because the light saturation point of the cucumber leaves changes with the light intensity inside the growth facility, we used an LED light source module set to 13 different light intensities: 20, 50, 100, 200, 300, 500, 700, 900, 1000, 1100, 1200, 1300 or 1500 μmol·m^−2^·s^−1^. We set the relative humidity (RH) inside to 50% using a humidity module. The Fluorescence option Flr Action at Log was set to 2: FoFm (dark) or FsFm’Fo’ (light), the Flash type was set to MultiPhase and the other parameters were set to default. Daily readings were taken at 08:30–11:30 and 14:30–17:30 hours to avoid the impact of crop “midday break” on the reliability of the data collected. In total, 312 groups of experiments were conducted. Three plants were randomly selected for measurement in each group to form a complete data set of 936 test samples.

## Model Construction Method

### Selection of core fluorescence parameters

The photosynthetic and fluorescence parameter data were measured for the newborn leaves using the multi-factorial test described. The correlation between each factor and the photosynthetic rate was analyzed, and the core input factor for the newborn leaf photosynthetic rate prediction model was selected.

First, to avoid the large dimensional differences between the sample data that would have a significant impact on the neural network training, the Tem, CO_2_ concentration, Par, RH, qP,

ETR, PhiPS2, Fv′/Fm′, NPQ, qN and other parameters were normalized. The normalization interval was [−1, 1], and the normalization formula is shown in Eq. (1).

1 $$y=2(x-{x}_{\min })/({x}_{\max }-{x}_{\min })-1$$

where y represents the normalized data; and ${x}_{\max }$ and ${x}_{\min }$ represent the maximum and minimum of the same dimension data sequence, respectively.

Using the normalized data, the Pearson correlation coefficient between each factor and the photosynthetic rate was calculated using SPASS^28^, a data processing software and the formulae in Eqs. (2, 3, 4 and 5).

2 $$r=\frac{{S}_{{x}_{j}y}}{{S}_{{x}_{j}}{S}_{y}}$$

3 $${S}_{{x}_{j}}=\sqrt{\mathop{\sum }\limits_{i=1}^{n}{({x}_{ij}-{\bar{x}}_{j})}^{2}/n}$$

4 $${S}_{y}=\sqrt{\mathop{\sum }\limits_{i=1}^{n}{({y}_{i}-\bar{y})}^{2}/n}$$

5 $${S}_{{x}_{j}y}=\frac{1}{n}\mathop{\sum }\limits_{i=1}^{n}({x}_{ij}-{\bar{x}}_{j})({y}_{i}-\bar{y})$$

where x and y represent the average values of ${X}_{ij}$ and ${y}_{i}$, respectively; ${x}_{j}$ (j = 1, 2, … 10) represents Tem, CO_2_, Par, RH, qP, ETR, PhiPS2, Fv′/Fm′, NPQ and qN; and y represents Pn. The correlation between each factor and the photosynthetic rate calculated from formula (2) is shown in Table 2.

### Construction of the photosynthetic rate prediction model

In this study, multi-factorial experiments were used to obtain multidimensional photosynthetic and fluorescent data, and key input factors were obtained through correlation and mechanism analyses. Then we programmed BP and PSO-BP methods based on MATLAB2015b software, without using any existing package. Among them, PSO-BP neural network was used to predict the photosynthetic rate of cucumber newborn leaves with environmental factors Tem, CO_2_, Par as input and net photosynthetic rate as output.

### Construction of the photosynthetic rate model using PSO-BP

**Figure 5:**
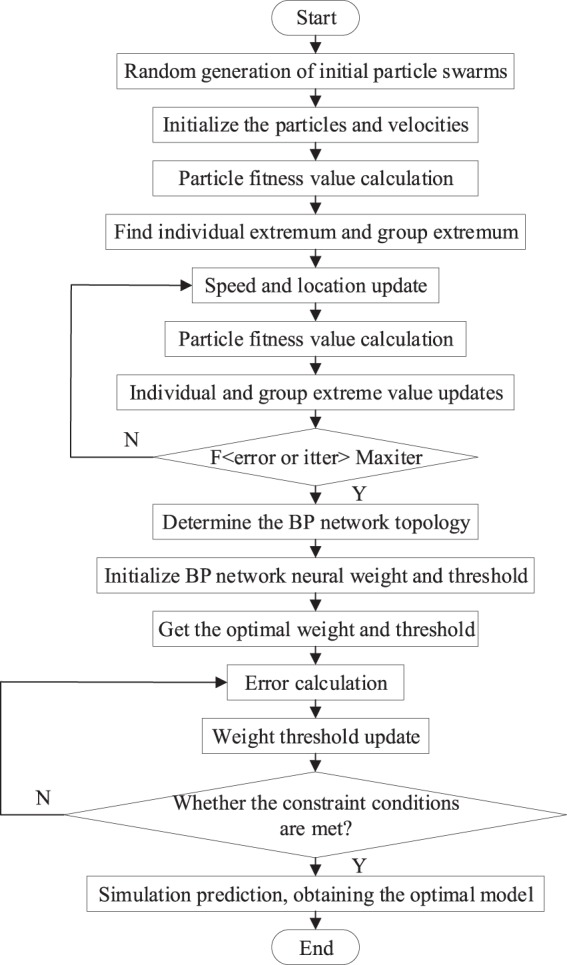
Flow chart for constructing the PSO-BP photosynthetic rate prediction model.

A fixed learning rate is used in traditional BP neural networks. However, this gives them many limitations, including slow network convergence, long training time and low local vulnerability^29,30^. By optimizing the initial weight of the network, the convergence speed and accuracy of the model can be improved. A BP neural network based on particle swarm optimization (PSO) provided a good foundation for the photosynthetic rate prediction model of the newborn leaves. A flow chart of the new leaf photosynthetic rate prediction model is shown in Fig. 5.

### BP neural network structure design

**Figure 6:**
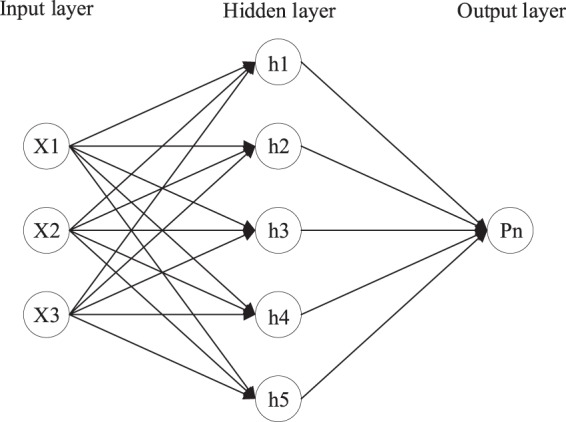
Basic network structure.

BP is a multi-layer feedforward network based on an error back-propagation algorithm^31–33^, and includes an input layer, hidden layer and output layer (Fig. 6). A single hidden layer structure was used to construct a photosynthetic rate prediction model using Tem, CO_2_, Par as three-dimensional input factors and the net photosynthetic rate as the output factor. The normalized input array was defined as X and the corresponding net photosynthetic rate, Pn, was defined as the output. The S-type tangent function Tansig was used as a hidden layer neuron transfer function, and the number of nodes is 5 which calculated in Eq. 6. The linear function Purelin was used for the output layer.

6 $$L=(m+n)/2+c$$

where m is the number of input layer nodes; n is the number of output layer nodes; and c is a constant between 1 and 10.

Among them, the hidden layer is mainly used to calculate the current output of a neuron according to its final state and total input^34^. The current output state of a neuron reflects its activity level, which is usually calculated by an activation function. In this paper, the S-type tangent function is ‘Tansig’, the formula is shown in Eq. 7:

7 $$f({\rm{X}})=\frac{1}{1+{e}^{-{\rm{X}}}}$$

Based on the output of the aforementioned neurons, the output layer calculates the output of the network^35^, which is, Pn′. It can be defined as an output function, which is usually a linear function without limiting amplitude. the formula is shown in Eq. 8:

8 $$Pn{\prime} ({\rm{X}})={\rm{X}}$$

Next, the error function is defined according to the output value of the network and the real value, which is used to update the weight of the network^36^. The formula is shown in Eq. 9:

9 $$E=\frac{1}{2}{\sum _{j}({{\rm{P}}{\rm{n}}}_{j}^{{\prime} }-{{\rm{Pn}}}_{j})}^{2}$$

where *E* represents the error function; Pn′ is the output value of the network; Pn is the real value; and *j* represents the *j*th neuron.

Based on the error function, the weight of the network is modified in the direction of e-function gradient^37^. the formula is shown in Eq. 10:

10 $$\Delta {w}_{ijk}=-\,\eta \frac{\partial E}{\partial {w}_{ijk}}$$

where ${w}_{ijk}$ represents the weight of the *j*-th neuron in the *k* − 1 layer connecting the *i*-th neuron in the *k*-th layer; $\Delta {w}_{ijk}$ represents the updated value of the weight; $\eta$ represents the step factor.

### PSO-BP network weights

To minimize error, BP learns and adjusts the network weights and thresholds through repeated training calculations. However, there are limitations to the training process where there are local minimums and large errors^33,38,39^. The PSO algorithm is derived from a simulation of bird predation behavior^40–42^. The fitness is used to measure the pros and cons of the particles and the optimal solution of the neural network is evaluated. In our study, we used the PSO algorithm to optimize the weight of the BP model. The initial population is 110, the fitness and position of the particles were updated by tracking using two extreme values. The optimal solution of particles is ${X}_{pbesti}$; that is, the individual extremum ${p}_{i}=({p}_{i1},{p}_{i2},\mathrm{..}.{p}_{ij})$. The optimal solution of the group is ${X}_{gbest}$, which is the Global extremum, $g=({g}_{1},{g}_{2},\mathrm{..}.{g}_{j})$. The particle speed and position update are shown in Eq. 11:

11 $$\{\begin{array}{c}{V}_{ij}=\omega {V}_{ij}+{c}_{1}rand()({P}_{ij}-{X}_{ij})+{c}_{2}rand()({P}_{gj}-{X}_{ij})\\ {X}_{ij}={X}_{ij}+{V}_{ij}\end{array}$$

where ${V}_{ij}$, ${X}_{ij}$ are velocity and position, respectively; $\omega$ is the inertia weight of the particle; rand() is a random function; ${P}_{ij}$ is the optimal value of the individual position; and ${P}_{gi}$ is the optimal value of the group position.

Based on the weight adjustment of the BP error algorithm, the PSO optimization algorithm was used to modify the weight. The global optimality was searched for using the PSO, and the global optimal solution was identified using the mean-square-error that is the fitness. The objective function is presented in Eq. 12:

12 $$E=\frac{1}{pL}\mathop{\sum }\limits_{i=1}^{p}\mathop{\sum }\limits_{j=1}^{L}{({T}_{oij}-{T}_{dij})}^{2}$$

where *p* is the number of samples; L is the number of neurons; ${T}_{oij}$ is the expected output value; and ${T}_{dij}$ is the predicted output value.

### Model training and validation

Data set based on the above experiments, 80% was used as the training set, and 20% was used as the validation set. The LM training method was used to train the model based on the PSO-BP neural network obtained. The entire set of leaf input samples was $X{\prime} ={[{X}_{1}^{{\prime} }{X}_{2}^{{\prime} }{X}_{3}^{{\prime} }]}^{T}$, in which ${X}_{1}^{{\prime} },{X}_{2}^{{\prime} },{X}_{3}^{{\prime} }$ were Tem, CO_2_ and Par, and the output signal, ${T}_{oij}$, represents the net photosynthetic rate calculated by the network. The corresponding measured net photosynthetic rate is ${T}_{d}$. When running the network, the optimal weight matrix obtained by PSO is received, and a set of sample sets $({X}^{\text{'}},{T}_{d})$ is the input that is used to construct the photosynthetic rate model for a different leaf position, ${T}_{d}^{\text{'}}({X}^{\text{'}})$.

## References

[1] Panda D (2019). Improvement of growth, photosynthesis and antioxidant defense in rice (oryza sativa l.) grown in fly ash-amended soil. Proceedings of the National Academy of Sciences, India Section B: Biological Sciences.

[2] Khazaei H, Wach D, Pecio A, Vandenberg A, Frederick L (2019). Stoddard. Genetic analysis of photosynthesis‐related traits in faba bean (vicia faba) for crop improvement. Plant Breeding.

[3] Tollenaar M (1989). Response of dry matter accumulation in maize to temperature: ii. leaf photosynthesis. Crop Science.

[4] Pantin F (2012). Thierry Simonneau & Bertrand Muller. Coming of leaf age: control of growth by hydraulics and metabolics during leaf ontogeny. New Phytologist.

[5] Trouwborst G, Sander WH, Harbinson J, Wim Van I (2011). The influence of light intensity and leaf age on the photosynthetic capacity of leaves within a tomato canopy. Journal of Pomology & Horticultural Science.

[6] Ran MY (2011). Acute exposure to uv-b sensitizes cucumber, tomato, and arabidopsis plants to photooxidative stress by inhibiting thermal energy dissipation and antioxidant defense. Journal of Radiation Research.

[7] Zvalinskii VI (2019). Quantitative modeling of photoacclimation and photoinhibition in marine phytoplankton. Oceanology.

[8] Wang N (2010). Antisense-mediated suppression of tomato zeaxanthin epoxidase alleviates photoinhibition of psii and psi during chilling stress under low irradiance. Photosynthetica.

[9] Zhang Y (2014). Estimation of vegetation photosynthetic capacity from space-based measurements of chlorophyll fluorescence for terrestrial biosphere models. Glob Chang Biol.

[10] Sáez PL, Rivera BK, Ramírez CF, Vallejos V, Bravo LA (2018). Effects of temperature and water availability on light energy utilization in photosynthetic processes of deschampsia antarctica. Physiologia Plantarum.

[11] Schedlbauer JL, Fetcher N, Hood K, Moody ML, Tang J (2018). Effect of growth temperature on photosynthetic capacity and respiration in three ecotypes of eriophorum vaginatum. Ecology & Evolution.

[12] Zhang Y, Guanter L, Joiner J, Song L, Guan K (2018). Spatially-explicit monitoring of crop photosynthetic capacity through the use of space-based chlorophyll fluorescence data. Remote Sensing of Environment.

[13] Antal T, Konyukhov I, Volgusheva A, Plyusnina T, Rubin A (2018). Chlorophyll fluorescence induction and relaxation system for the continuous monitoring of photosynthetic capacity in photobioreactors. Physiologia Plantarum.

[14] Yin, G., Zhao, N., Shi, C., Chen, S. & Liu, W. Phytoplankton photosynthetic rate measurement using tunable pulsed light induced fluorescence kinetics. *Optics Express* **26**, A293–A300 (2018). 10.1364/OE.26.00A293 29609388

[15] Hong SJ, Zhang Y, Yin L, Wei L, Huang W (2018). Diurnal changes in photosynthesis by six submerged macrophytes measured using fluorescence. Aquatic Botany.

[16] YAN Li, DING Yunjie, LIU Jia, ZHU Hejun, LIN Liwu (2011). Influence of Phosphine Concentration on Propylene Hydroformylation over the PPh3-Rh/SiO2 Catalyst. Chinese Journal of Catalysis.

[17] Gao Y, Gao ZK, Zhang XH, Gao RF (2009). Heat shock stress on photosystem II in while cucumbers probed by the fast fluorescence rise OJIP. Acta ecologica sinica..

[18] Zhang, H., Tao, Y. & Hu, J. Photosynthetic rate prediction model of cucumber seedlings fused chlorophyll content. *Transactions of the Chinese Society for Agricultural Machinery* **46**, 259–263 and 307 (2015).

[19] Yin J, Liu X, Zhang M, Han LI (2017). Photosynthetic Rate Prediction of Tomato under Greenhouse Condition in Spring and Autumn Growth Period. Transactions of the Chinese Society for Agricultural Machinery..

[20] Urban O (2018). Combined effects of drought and high temperature on photosynthetic characteristics in four winter wheat genotypes. Field Crops Research.

[21] Shi, D. W., Wei, X. D. & Chen, G. X. Effects of low temperature on photosynthetic characteristics in the super-high-yield hybrid rice ‘Liangyoupeijiu’ at the seedling stage. *Genetics and molecular research: GMR* **15** (2016). 10.4238/gmr15049021 27966747

[22] Wattal, R. K. & Siddiqui, Z. H. Effect of elevated levels of carbon dioxide on the activity of rubisco and crop productivity. (Springer International Publishing, 2015).

[23] Pettersson R, Mcdonald AJS (2006). Effects of elevated co2 growth and photosynthesis on small birch plants (betulapendula roth.). Plant Cell & Environment.

[24] Zhou WL, Liu WK, Yang QC (2012). Quality changes in hydroponic lettuce grown under pre-harvest short-duration continuous light of different intensities. Journal of Horticultural Science & Biotechnology.

[25] Hari P, Luukkanen O (2006). Field studies of photosynthesis as affected by water stress, temperature, and light in birch. Physiologia Plantarum.

[26] Kitao M (2003). Light-dependent photosynthetic characteristics indicated by chlorophyll fluorescence in five mangrove species native to Pohnpei Island, Micronesia. Physiol. Plant..

[27] Jablonski A, Kruger EL, Townsend PA (2017). Comparative responses of solar-induced fluorescence (SIF) and leaf photosynthetic parameters to short term atmospheric CO2 enrichment. AGUFM.

[28] Li Mingxing, Zhang Mengjuan, Yuan Jun, Zhao Mengyuan, Zhang Min (2015). The correlation analysis of patent output and economic efficiency in intellectual property rights intensive industries. Journal of Interdisciplinary Mathematics.

[29] Liu J, Qiu X (2009). A Novel Hybrid PSO-BP Algorithm for Neural Network Training. in 2009 International Joint Conference on Computational Sciences and Optimization.

[30] Ding S, Wu QH (2013). A matlab-based study on approximation performances of improved algorithms of typical bp neural networks. Applied Mechanics & Materials.

[31] Wang B, Gu X, Li M, Yan S (2016). Temperature error correction based on bp neural network in meteorological wireless sensor network. International Journal of Sensor Networks.

[32] Liu T, Shao J (2018). Simulation of soil erosion intensity in the three gorges reservoir area using bp neural network. Journal of Natural Resources.

[33] Tan X, Ji Z, Zhang Y (2018). Non-invasive continuous blood pressure measurement based on mean impact value method, bp neural network, and genetic algorithm. Technology & Health Care Official Journal of the European Society for Engineering & Medicine.

[34] Yu, Y., Fu, Y. & Wu, X. Metric and classification model for privacy data based on shannon information entropy and bp neural network. *Journal on Communications,*10.11959/j.issn.1000-436x.2018286 (2018).

[35] Xiao J, Liu S, Hu L, Wang Y (2018). Filtering method of rock points based on BP neural network and principal component analysis. Front. Comput. Sci..

[36] Chen S, Xie X, Zheng F, Wu S (2019). Auto focusing method of imaging system of digital pcr instrument based on bp neural network. International Journal of Pattern Recognition and Artificial Intelligence.

[37] Lu Y, Zhang PP, Wang XY, Wang H, Zhao CX (2017). Aacmm length error compensation based on pso-bp neural network. Acta Metrologica Sinica.

[38] Hou C, Xiao Y, Cao Y, Lai C, Cao Y (2018). Prediction of synchronous closing time of permanent magnetic actuator for vacuum circuit breaker based on pso-bp. IEEE Transactions on Dielectrics & Electrical Insulation.

[39] Liu P, Zhang W (2018). A fault diagnosis intelligent algorithm based on improved bp neural network. International Journal of Pattern Recognition and Artificial Intelligence.

[40] Nammalvar P, Ramkumar S (2018). Parameter improved particle swarm optimization based direct-current vector control strategy for solar pv system. Advances in Electrical and Computer Engineering.

[41] Chou LD, Chen HF, Tseng FH, Chao HC, Chang YJ (2018). Dpra: dynamic power-saving resource allocation for cloud data center using particle swarm optimization. IEEE Systems Journal.

[42] Phoemphon S, So-In C, Niyato D (2018). A hybrid model using fuzzy logic and an extreme learning machine with vector particle swarm optimization for wireless sensor network localization. Applied Soft Computing.

